# Patients' Views on a Combined Action Observation and Motor Imagery Intervention for Parkinson's Disease

**DOI:** 10.1155/2016/7047910

**Published:** 2016-09-29

**Authors:** Judith Bek, Jordan Webb, Emma Gowen, Stefan Vogt, Trevor J. Crawford, Matthew S. Sullivan, Ellen Poliakoff

**Affiliations:** ^1^University of Manchester, Manchester, UK; ^2^Lancaster University, Lancaster, UK; ^3^Manchester Metropolitan University, Manchester, UK

## Abstract

*Background*. Action observation and motor imagery activate neural structures involved in action execution, thereby facilitating movement and learning. Although some benefits of action observation and motor imagery have been reported in Parkinson's disease (PD), methods have been based on stroke rehabilitation and may be less suitable for PD. Moreover, previous studies have focused on either observation or imagery, yet combining these enhances effects in healthy participants. The present study explores the feasibility of a PD-specific home-based intervention combining observation, imagery, and imitation of meaningful everyday actions.* Methods*. A focus group was conducted with six people with mild to moderate PD and two companions, exploring topics relating to the utility and feasibility of a home-based observation and imagery intervention.* Results*. Five themes were identified. Participants reported their experiences of exercise and use of action observation and motor imagery in everyday activities, and the need for strategies to improve movement was expressed. Motivational factors including feedback, challenge, and social support were identified as key issues. The importance of offering a broad range of actions and flexible training was also highlighted.* Conclusions*. A home-based intervention utilising action observation and motor imagery would be useful and feasible in mild to moderate PD.

## 1. Introduction

People with PD often have difficulty in initiating movements, and movements can be slower and reduced in size. External cueing can be effective in facilitating movement in PD [1, 2]; for example, lines on the floor or laser pointers can help to improve initiation of gait and maintain appropriate step length. Observation of human movement may provide another form of cueing, and researchers have recently begun to investigate the therapeutic potential of action observation (AO) in rehabilitation for PD (for reviews see [3, 4]).

Observing another person's movement activates neural structures involved in performing the same movement [5, 6], facilitating subsequent action. Imitation involves both the observation and execution of an action, thereby offering a particularly effective means of priming movement [7, 8], while motor imagery (MI) is the imagination of a movement in the absence of action execution. MI involves visual and kinaesthetic (sensorimotor) representations and also activates the motor system [9, 10]. AO, MI, and imitation have been shown to improve movement and increase learning in healthy individuals (e.g., [11–13]).

Previous research has shown benefits of AO and MI in stroke rehabilitation, with improvements in activities of daily living found after training with AO and imitation [14–16], as well as MI interventions [17]. Increased neural activation in the motor system has also been reported following AO training in stroke [14]. The evidence base documenting the effects of such interventions in PD is comparatively sparse, although some encouraging results have been reported. For example, AO has been found to improve sequential movements, increase self-reported mobility, and reduce freezing of gait [18–20], and MI has also been shown to reduce freezing of gait [21]. Although no large-scale studies of AO or MI training have been reported in PD, a pilot randomised controlled study found improvements in functional independence following training with AO and imitation alongside conventional physiotherapy, compared with physiotherapy alone [22]. Training with MI has also been found to improve timed motor performance when combined with physical practice [23].

Although imitation ability may be affected in PD [24, 25], the above findings suggest that it can nonetheless be applied successfully in rehabilitation. MI has also been shown to be preserved in PD [26–28], although compensatory mechanisms may be involved [28, 29]. Previous studies in PD have focused on either AO or MI, but behavioural [12, 30] and neuroimaging [31–33] findings indicate that combining these processes can enhance effects and increase activation of the motor system in healthy individuals (for review, see [34]). Based on these findings, we sought to obtain patients' views on the development of a PD-specific intervention utilising principles of AO, MI, and imitation.

Importantly, while benefits of AO interventions for everyday actions have been demonstrated in stroke patients, different objectives must be considered in designing a PD-specific intervention. Whereas stroke rehabilitation aims to restore or compensate for lost function, therapies for PD should be targeted at improving or maintaining control of movement, such as facilitating initiation and increasing amplitude or speed. Thus, it is important to consult with people with PD and those working with PD patients to identify appropriate training goals, as well as consulting potential users on issues relating to feasibility and design of interventions [35, 36]. An initial concept for an AO + MI intervention for PD has been informed by researchers with expertise in PD and AO, as well as physiotherapists, clinicians, and PD representatives. The proposed intervention will be delivered via mobile technology in the user's home (using a tablet PC, although other devices could potentially also be used), with suggested daily practice of approximately 30 minutes, allowing flexibility to accommodate other commitments and fluctuations in symptoms. Training consists of observing videos of actions, engaging in motor imagery, and then performing the target action. Users will be provided with a set of personally selected training videos, working with a researcher or therapist to devise an individualised training programme. The aim of the present study was to conduct a focus group to explore the views of people with PD and their carers or companions on the proposed intervention.

## 2. Method

Ethical approval for the study was obtained from a UK National Health Service Research Ethics Committee (NRES Committee North West, Greater Manchester South), and written informed consent was obtained from all participants.

### 2.1. Participants

Participant demographics are reported in Table 1. Six people with idiopathic PD (4 males and 2 females) presenting with mild to moderate symptoms (Hoehn & Yahr stages 1–3 [37]) were recruited from a panel of research volunteers who had taken part in previous studies at the University of Manchester. All but one of the participants were taking dopaminergic medication. Companions (1 male and 1 female) of two of the PD participants (spouses of P2 and P5, resp.) also contributed to the focus group discussions. The present sample size is typical of focus group studies and based on recommendations in the literature [38].

### 2.2. Procedure

Demographic information and disease history were obtained prior to the session, and motor examination scores from the Unified Parkinson's Disease Rating Scale (UPDRS [39]) were obtained within a period of 8 weeks prior to the focus group as part of a previous study. Participants also completed a short form of the Parkinson's Disease Questionnaire (PDQ-8 [40]) on the day of the focus group to provide a general measure of functioning and well-being.

To assess the ability of participants to engage in motor imagery, we analysed data collected as part of a previous study using a short form of the Kinaesthetic and Visual Imagery Questionnaire (KVIQ [41]). The KVIQ was devised to measure MI in people both with and without disabilities and has been validated for use in PD patients [42]. Participants are asked to perform and then imagine performing simple movements of the head, shoulders, trunk, upper limbs, and lower limbs. The clarity of the image (visual imagery) and intensity of the sensations (kinaesthetic imagery) are rated on a 5-point scale, with higher scores representing stronger imagery.

The focus group took place at the University of Manchester and lasted approximately 1 hour and 20 minutes. The session was chaired by one researcher and facilitated by two others, and a schedule of topics was used to guide discussions. Open-ended questions were used with prompts to elicit further information where needed, and visual aids were used to help explain concepts underlying the proposed therapy.

Participants were first asked about their general experience of physical activity or physiotherapy and their thoughts on a home-based intervention to improve movement. The concepts of action observation, motor imagery, and imitation were then introduced (some advance information had also been provided in a participant information sheet) and participants were asked if they had any experience of using these or other strategies to manage symptoms or to facilitate movement. The proposed intervention was then introduced, and participants were shown example videos depicting potential actions to be trained (e.g., cleaning glasses). Further discussions centred on identification of actions that would be useful and relevant to include in training, as well as practical issues such as intensity and timing of training and the use of technology.

Participants were advised that any comments made during the focus group could subsequently be removed from the analysis if they so wished. Responses were digitally recorded and transcribed verbatim by an independent transcription service.

### 2.3. Data Analysis

Data were analysed using thematic analysis, which is a flexible method of identifying and analysing patterns within a dataset [43]. A primarily deductive approach to analysis was taken to identify themes relating to feasibility and potential target actions, while also allowing for data-driven identification of themes.

The data were coded by the second author (J. Webb) and categories were identified, which were organised into themes. The transcript was then reanalysed by the first author (J. Bek) using the categories and themes identified. Final themes were verified following further discussion within the research team.

## 3. Results

Motor imagery scores for both visual and kinaesthetic subscales of the KVIQ (Table 1) were within the range previously reported for healthy individuals of a similar age [41], indicating that participants were able to engage in motor imagery and that vividness of imagery was similar to that of healthy older adults.

Analysis revealed five themes, which are described below.

### 3.1. Theme 1: Experience of Exercise/Physiotherapy and Its Benefits

Participants reported benefits obtained from physiotherapy and exercise that they had previously undertaken, such as gym training.

*I go to the gym very often…exercise does help.*  (P5)

### 3.2. Theme 2: Use of AO and MI in Everyday Activities

#### 3.2.1. Subtheme 2.1: Action Observation/Imitation

Participants related experiences of using action observation and imitation in the context of exercise classes or in the gym and when walking:

*I do quite a lot of pilates and yoga classes which are classic examples of imitating somebody else to do the right movements.* (P4)

*I do find it easier when I'm mimicking walking with [husband] and I tend to walk the same as he does.* (P2)

Participants were also aware of experiencing “automatic” imitation in which observed behaviours and gestures are unconsciously mimicked in social contexts [44]:

*I do tend to mimic people when I'm talking. I tend to copy their hand movements in socialising.* (P2)

#### 3.2.2. Subtheme 2.2: Motor Imagery

Participants reported using motor imagery in sports and leisure pursuits (racquetball and playing the piano) as well as in more basic movement contexts (getting into bed and walking through doorways):

*The way I get into bed often is to sit and then spring both my legs up to be along the length of the bed and I guess am thinking quite consciously about how I'm going to do that.* (P3)

*…the physiotherapist recommended imagining…if I could remember to do it then I could walk straight through a doorway.* (P6)

In addition to the use of MI as a cue to action, participants reported mentally rehearsing activities outside of the immediate context:

*I took up the piano again…if I'm trying to get to sleep I kind of play through some of my pieces and I think that kind of mental reinforcement definitely helps the physical agility.* (P4)

### 3.3. Theme 3: Indication of Need for Strategies to Improve Movement

#### 3.3.1. Subtheme 3.1: Interest in Alternative Ways to Manage Symptoms

Participants expressed an interest in methods to help them to improve movement or better manage their symptoms:

*…spent the first three years resisting taking any drugs, so I was really focusing on movement and exercise to keep me going. *(P4)

*I'm really interested in ways of managing movement that might allow for a reduction in drugs. *(P6)

*…with a tool like this, you could have the potential to capture data and develop a numerical score that might be helpful in therapy perhaps.* (P3)

#### 3.3.2. Subtheme 3.2: Use of Compensatory Strategies

Participants reported strategies they had used to compensate for their difficulties in performing everyday tasks:

*So if my left hand was playing up, I'd sit on it and just practise with my right hand.* (P4)

*…five finger typing. I long ago reduced to one hand and now even that's getting a bit of a problem. My main solution to that is to talk to the computer and that is very good. I've got a program … *(P3)

There was also discussion surrounding “the dilemma…whether you put your energy into practice, or finding alternatives or more convenient and effective methods” (P4):

*…brushing your teeth, I found I was holding my brush and moving my head. I have avoided that by getting an electric toothbrush. Perhaps I should have carried on just using a brush.* (P3)

*…if I do a lot of writing, it is really uncomfortable, but my consultant says to not stop writing, just to keep doing it.* (P1)

### 3.4. Theme 4: Actions and Aspects of Movement

Participants discussed everyday actions that they had difficulty with and would find useful to train (see Table 2), and it was clear that requirements varied between individuals.

#### 3.4.1. Subtheme 4.1: Everyday Actions

The majority of suggested actions were everyday activities relating to personal care, dressing, work/leisure activities, and household chores. The difficulties participants reported with these actions typically involved fine motor coordination and dexterity:

*…trying to do things quickly with the mouse buttons, that's awkward*. (P1)

*…to enhance my ability even just with the one hand to type would be a big help.* (P3)

*I wear contact lenses…cleaning those is really difficult to put the solution on and rub my two fingers together.* (P1)

*I can't believe how bad I am at folding laundry…it seems the most difficult thing in the world… *(P4)

Other suggestions indicated the desire to improve more basic mobility tasks, in relation to hypokinesia, (*…very little steps…I could imagine that that could be improved* (P3)) and difficulties with action initiation (*Going upstairs, sometimes you freeze and you just can't get started* (P2)).

#### 3.4.2. Subtheme 4.2: Core Components of Movement

There was also discussion surrounding the value of training specific functional actions versus core movement skills that could be applied more broadly:

*…what would be interesting to me would be to see whether there's any way of refining core skills so that those skills could then translate into specific contexts…be it brushing teeth or making a cup of tea…the exercises that you do wouldn't necessarily be to do with that but would actually reinforce the skills that you could then apply to whatever the particular task is… *(P4)

*Having taken up yoga classes, my neck movements have improved enormously and I'm getting the benefit of it when I reverse the car… *(P4)

### 3.5. Theme 5: Feasibility

This theme encompassed issues pertaining to the feasibility of the proposed intervention, with categories relating to motivation, choice/flexibility, and practical issues.

#### 3.5.1. Subtheme 5.1: Motivation

Comments from all participants indicated that motivation was a key factor in engaging with rehabilitation. In particular, participants wanted to feel that they were progressing in their training:

*…it's got to be motivating, doing something purely because it will get better, they tell me, or my coach tells me, whatever, would be not enough, I'd want to experience something beneficial to me happening.* (P3)

The potential impact of apathy was also noted:

*…apathy is a significant feature of Parkinson's…so it's actually harder to sustain things.* (P4)

The need to feel challenged was indicated by some participants:

*…the actions were so basic and simple that it was hard to feel interested or motivated in them.* (P6)

However, an appropriate level of difficulty was considered important in order to build confidence and maintain engagement in training:

*…if you can't do something, it's just hopeless because you can't build on it, can you? And if it's too easy, that's hopeless too because there's no reward to it. It's got to be exactly at the right level for the individual*. (P4)

Comments indicated participants' desire to receive feedback on their performance and progress. This might be through noticeable improvement in the trained actions but could also involve a system of awarding scores or points for progress:

*…to give me the immediate feedback that it is beneficial now, I can clean my teeth better or I can hit the keyboard better or something*. (P3)

*…you're moving onto the next level or whatever, those sorts of little motivational tricks that are everywhere in computer games now.* (P4)

Corrective feedback was also considered important:

*…if you're not achieving your goals, you need someone to explain what you need to do to try and improve.* (P1)

Motivation was also discussed in relation to the intensity of training. While some participants indicated that they would not be able to commit to the suggested 30 minutes of daily training, others felt that this would be achievable:

*…getting the motivation to do it every day for half an hour might be difficult. *(P3)

*I think I'd do it a couple of times a week, otherwise I'd think I'd probably be down to ten minutes a day, I'd love to do it but I can only do ten minutes a day.* (P4)

*I think personally for me that would be ideal, but not necessarily for somebody else. *(P2)

The importance of social factors in motivation to engage in training was also highlighted, with participants suggesting a group setting or online social support:

*Personally I prefer to go out to a class and do it, to be interactive with other people*. (P1)

*…it's rewarding to talk to other likeminded people*. (P2)

*I think if there was an online group …something so that you're not doing it in isolation*. (P4)

#### 3.5.2. Subtheme 5.2: Choice and Flexibility

It was considered important for individuals to be able to choose from a broad range of actions:

*…a package that had the most range of options from very simple to more complex…The more you can build into something, the more likely people are to have a go at part of it, I would think*. (P6)

Comments indicated that the difficulty of a particular action may be variable within an individual from day to day, further emphasising the need for flexibility in training:

*…sometimes it's easy to manoeuvre a mouse or use a keyboard, other days it's near on impossible.* (P5)

It was also suggested that the proposed intervention could be applied to different settings:

*It's an additional resource, isn't it, and it can be used by individuals but it can also be used by people supporting people with Parkinson's and through Parkinson's groups. It doesn't have to be just one on one*. (P4)

#### 3.5.3. Subtheme 5.3: Practical Considerations

Possible limitations were identified in terms of age, motor impairment, and the use of technology:

*I guess it's no good to have an iPad if you can't get into it because your finger isn't…you sort of fail on the first hurdle, don't you?* (P4)

*…the population with Parkinson's that's older than 70 is huge, and some of them might well be interested in doing something but not as able or as experienced at using technology.* (P6)

However, participants felt that the use of mobile technology would be acceptable and might be particularly useful in certain groups:

*I'm sure there would be people that would use it and get benefit out of an app on an iPad*. (P1)

*…something delivered on an iPad would be particularly helpful, I'd have thought, for people in rural areas where there's an issue of transport and mobility.* (P4)

## 4. Discussion

Analysis of the focus group discussions indicated that people with mild to moderate PD perceived physical activity to be beneficial, as reported previously [35, 45], and were also able to relate positive experiences of using action observation and motor imagery in their daily activities and management of their symptoms.

A need to identify strategies to improve control of movement was expressed. This is consistent with the findings of another recent focus group study, in which the importance of physical approaches to symptom management was reported as an unmet need in the healthcare of people with PD, including access to home activity programmes and management of daily activities [46]. Moreover, the proposed intervention might allow people to continue to perform tasks as they have done previously, instead of finding alternative compensatory strategies.

Potential targets for training were then discussed. Participants reported difficulties in performing a range of everyday actions, which related to symptoms of PD such as bimanual coordination, hypokinesia, and initiation. The heterogeneity of suggested actions indicates variation among individuals with PD in the actions that they find difficult or would value the opportunity to improve. Preferences for training core components of movement or specific tasks also differed between participants. Since some individuals may already be using compensatory strategies for particular tasks, it may be beneficial to offer different options within action observation training (i.e., viewing the task performed using either the original method or the compensatory technique).

Motivation was identified as a key factor in the feasibility of the proposed intervention, consistent with previous findings on patients' views on exercise [35, 45]. Home-based training increases independence and choice for therapy users, as recommended by UK NICE guidelines [47], and independent exercise programmes have previously shown positive effects in PD [48, 49]. However, apathy is a prevalent symptom in PD [50] and its potential impact on adherence to training was considered. Discussions indicated that an appropriate level of difficulty or challenge, monitoring of progress, feedback, and social interaction are all potential contributors to the successful implementation of a user-led home-based intervention. These issues are also of broader relevance in designing interventions for PD and other neurological conditions, and have been identified previously in relation to the development of video-game based therapies [51, 52].

Choice and flexibility were also highlighted as important considerations in the design of home training programmes. Participants expressed the desire to be offered a broad range of actions to train. Personalised action observation therapy, using actions meaningful to the individual, has been found to increase independence in stroke patients [53], and this approach is consistent with Parkinson's UK “personalised treatments” research priority [54]. Quinn et al. [35] have also highlighted the importance of the patient taking an active role in the design of rehabilitation programmes. Symptoms can fluctuate from day to day and also over the course of the day in PD, and training programmes should accommodate this variation in terms of the duration and intensity of practice, which could be selected by the user according to their symptomatic status. Moreover, some users in earlier disease stages may still be in employment or have domestic responsibilities; flexibility is therefore also important in allowing users to adapt their training to fit with their lifestyle.

Finally, practical considerations were highlighted, including the potential difficulty in using mobile technology to deliver training. There is increasing recognition of the value of technology, such as smartphones and tablet PCs, in diagnosis, disease monitoring, and therapy in PD (e.g., [55, 56]). Neurorehabilitation approaches based on video gaming and virtual reality have been implemented successfully in PD [51, 52], and a recent study demonstrated feasibility of a tablet PC-based action observation therapy for stroke patients [57]. However, the present study indicates the importance of considering the effects of age and motor impairment on the use of such technology by some individuals with PD.

Participants' self-reported vividness of MI was similar to that previously found in healthy older adults [41]. This is consistent with other studies showing intact MI in PD [26–28], suggesting that people with PD would be able to engage in MI-based training. However, the proposed intervention may be suitable only for a subset of people with mild to moderate PD who are motivated and able to train at home. AO + MI training may also be best utilised as a tool to complement traditional physiotherapy or group exercise classes, and support from therapists or via a social network may increase uptake of and adherence to training. Although we cannot extrapolate from our findings to patients with more advanced PD, this population may nonetheless also be able to benefit from an AO + MI intervention. Individuals who are not able to physically perform the movements may still be able to practice using AO and MI, but alternative delivery methods might need to be considered.

While providing valuable information to guide the development of an AO + MI intervention for PD, the present study is limited by its small sample size. The participants in the present study were proactive and motivated research volunteers and may thus be unrepresentative of the target population. In addition, although motor imagery ability was intact in the present sample, some individuals with PD may have difficulty in engaging successfully in imagery [26]. Further consultation with carers or family members and healthcare professionals (e.g., physiotherapists and occupational therapists) is also needed to inform intervention development.

## 5. Conclusions

The focus group indicated that people with mild to moderate PD considered a home-based action observation and motor imagery intervention to be useful and feasible. Key issues in designing home-based interventions were identified, including motivation and choice. The study also highlighted the importance of consulting with user groups in intervention development.

## Figures and Tables

**Table 1:** Participant demographics and background assessments.

Participant	Sex	Age (years)	Disease duration (years)	Hoehn & Yahr stage	UPDRS motor score	PDQ-8	KVIQ-V	KVIQ-K
1	M	64	2.2	1	26	12.5	17	8
2	F	56	8.5	1	27	40.6	17	13.5
3	M	69	6	2	31	21.9	18.5	19
4	F	63	2.8	3	43	15.6	22.5	18
5	M	73	1	3	63	15.6	14	16
6	M	60	20	3	64	43.8	15	12.5

PDQ-8 = Parkinson's Disease Questionnaire; score given as a percentage (higher scores indicate poorer health status). KVIQ = Kinaesthetic and Visual Imagery Questionnaire (V = visual subscale and K = kinaesthetic subscale); scores out of a maximum of 25.

**Table 2:** Everyday actions suggested for inclusion in a home-based training programme.

Work/leisure activities	Household chores	Personal care	Dressing	Mobility
Using computer mouse Typing Writing Separating newspaper pages Folding newspapers	Folding laundry Opening garbage bags/grocery bags Cutting vegetables	Cleaning teeth Shaving Cleaning contact lenses	Putting on sweater/shirt/coat Putting on socks/tights Fastening buttons	Walking (step length) Climbing stairs
